# Effects of an Oxycodone Conjugate Vaccine on Oxycodone Self-Administration and Oxycodone-Induced Brain Gene Expression in Rats

**DOI:** 10.1371/journal.pone.0101807

**Published:** 2014-07-15

**Authors:** Marco Pravetoni, Paul R. Pentel, David N. Potter, Elena H. Chartoff, Laura Tally, Mark G. LeSage

**Affiliations:** 1 Minneapolis Medical Research Foundation, Minneapolis, Minnesota, United States of America; 2 University of Minnesota, School of Medicine, Department of Medicine, Minneapolis, Minnesota, United States of America; 3 University of Minnesota, School of Medicine, Department of Pharmacology, Minneapolis, Minnesota, United States of America; 4 Harvard Medical School, Department of Psychiatry, McLean Hospital, Belmont, Massachusetts, United States of America; The Scripps Research Institute, United States of America

## Abstract

Prescription opioid abuse is an increasing public health concern in the USA. A vaccine comprising a hapten (OXY) conjugated to the carrier protein keyhole limpet hemocyanin (OXY-KLH) has been shown to attenuate the antinociceptive effects of oxycodone. Here, the vaccine's ability to prevent acquisition of intravenous (i.v.) oxycodone self-administration was studied in rats. Effects of vaccination on oxycodone-induced changes in the expression of several genes within the mesolimbic system, which are regulated by chronic opiate use, were also examined. Vaccination with OXY-KLH reduced the proportion of rats acquiring i.v. self-administration of oxycodone under a fixed ratio (FR) 3 schedule of reinforcement compared to control rats immunized with the unconjugated KLH carrier protein. Vaccination significantly reduced the mean number of infusions at FR3, total number of infusions, and total oxycodone intake during the entire protocol. Compared to oxycodone self-administering control rats immunized with the carrier alone, rats vaccinated with the OXY-KLH immunogen showed increased levels of adenylate cyclase 5 (Adcy5) and decreased levels of early growth response protein 2 (Egr2) and the early immediate gene c-Fos in the striatum. These data suggest that vaccination with OXY-KLH can attenuate the reinforcing effects of oxycodone at a clinically-relevant exposure level. Analysis of mRNA expression identified some addiction-relevant markers that may be of interest in understanding oxycodone effects or the protection provided by vaccination.

## Introduction

Current pharmacotherapies for treatment of opioid addiction are effective, but show side effects and limitations. New strategies to meet the therapeutic challenge of treating opioid addiction are needed [1]. Active immunization with conjugate vaccines has been studied as a complementary option for the treatment of drug addiction [2].

Early studies of morphine conjugate vaccines showed that immunization attenuated heroin self-administration in non-human primates, decreased morphine brain distribution and attenuated morphine analgesia in mice and rats [3]–[5]. Development of immunotherapy strategies for treatment of opioid addiction was abandoned in the 70's, presumably because of Food and Drug Administration (FDA) approval of methadone replacement therapy, which quickly became the main form of treatment for opioid addiction [6]. More recent reports have shown that immunization with heroin or morphine conjugate vaccines, containing haptens based on morphine or 6-acetlymorphine, decreases brain distribution of the primary active heroin metabolite 6-acetylmorphine and attenuates heroin and morphine self-administration in rats [7]–[12]. These vaccines have not yet reached clinical trials.

Prescription opioids such as oxycodone and hydrocodone have high abuse liability, are widely prescribed as analgesics, and are readily accessible to teens and young adults, all of which are factors that have led to their increased abuse [13]. To address prescription opioid abuse, our laboratory developed a vaccine (OXY-KLH) that elicits the production of high titers of serum antibodies that bind oxycodone and hydrocodone in serum, prevents early distribution of clinically-relevant doses of oxycodone and hydrocodone to brain, and blocks oxycodone and hydrocodone antinociception in mice and rats [14]–[17]. The purpose of the present study was to test whether vaccination with OXY-KLH would prevent the acquisition of oxycodone self-administration in rats, an addiction-relevant behavior, in order to assess its efficacy in a pre-clinical model of prescription opioid abuse. Oxycodone self-administration has been previously characterized in animal models of addiction and reward [18]–[22]; however no studies have yet addressed the effects of vaccination against oxycodone in these models.

A secondary and exploratory purpose of the present study was to characterize the extent to which vaccination with OXY-KLH mitigates some of the neural changes induced by repeated oxycodone exposure. For instance it has been shown that oxycodone self-administration increases striatal dopamine levels [22]. A number of genes have been identified in brain regions implicated in mediating addiction, most notably the striatum, which are up- or down-regulated in response to repeated or persistent opioid exposure. Changes in brain gene expression have been reported after repeated experimenter-administered oxycodone in rats [23], [24] or after oxycodone self-administration in mice [20], [21]. These more recent studies showed that oxycodone self-administration alters gene expression in various neurotransmitter systems in the mouse dorsal striatum [20], [21].

Vaccination with OXY-KLH reduces brain concentrations of oxycodone in rats after a single oxycodone dose that elicits near maximal antinociception in the hot plate test [14]. It is not clear if this acute reduction in oxycodone distribution to the brain is sufficient to protect against repeated drug-induced changes in gene expression that may be involved in mediating reward and addiction. Evaluating gene expression during the acquisition of oxycodone self-administration in vaccinated rats affords an opportunity to address this question in a behaviorally relevant context.

In the current study, we investigated the effects of vaccination on the acquisition of oxycodone self-administration in rats. For exploratory purposes, after completion of the oxycodone self-administration study, we also evaluated the effects of vaccination on mRNA expression of opioid receptors and their downstream effectors in the nucleus accumbens (NAc) and caudate putamen (CPu) brain regions, which are key structures in the reward pathway.

## Materials and Methods

### 2.1 Ethics statement

All studies were approved by the Minneapolis Medical Foundation Care and Use Committee (protocol #08–10). All animals were euthanized by CO_2_ inhalation using AAALAC approved chambers, and efforts were made to minimize suffering and discomfort.

### 2.2. Animals

All animal studies were approved by the Minneapolis Medical Research Foundation Institutional Animal Care and Use Committee. Male Holtzman rats (300–324 grams at arrival) were purchased from Harlan (Indianapolis, IN), and housed in a temperature- and humidity-controlled colony room on a 12/12 hours dark/light inverted schedule. Rats had unlimited access to water, but were food restricted to 18 grams per day of standard rat chow.

### 2.3 Drugs and reagents

Oxycodone was obtained from Sigma (St. Louis, MO) and doses are expressed as the weight of the base.

### 2.4 Hapten synthesis and conjugation to carrier protein

An oxycodone hapten containing a tetraglycine linker at the C6 position (OXY) was first synthesized and then conjugated to chicken ovalbumin (OVA) for ELISA assays or to the keyhole limpet hemocyanin (KLH) carrier protein for vaccination studies as previously described [14], [15].

### 2.5 Serum antibody titers and concentrations

ELISA 96-well plates were coated with OXY-OVA conjugate or unconjugated OVA control in carbonate buffer and blocked with 1% gelatin. Primary antibodies were incubated with goat anti-rat IgG antibodies to measure oxycodone-specific serum IgG antibody titers, as described previously [15]. Rat oxycodone-specific serum IgG concentrations were measured by ELISA using a standard curve constructed using commercially available murine monoclonal anti-oxycodone IgG (Qed Biosciences, San Diego, CA), as previously described for anti-morphine IgG concentrations [25].

### 2.6 Vaccination

Rats received the OXY-KLH conjugate vaccine or a control vaccination consisting of the unconjugated KLH carrier protein alone (n = 12 each group). Rats were immunized i.p. with either 100 µg of each immunogen on days 0, 21, 42 and 63 using Freund's Complete Adjuvant for the first vaccination and Freund's Incomplete Adjuvant for all subsequent boosts as previously described [15]. A week after the last immunization, tail vein blood samples were collected to analyze oxycodone-specific serum antibody titers and concentrations by ELISA. Rats were then implanted with jugular vein indwelling catheters as previously described [26].

### 2.7 Apparatus

Sixteen identical operant conditioning chambers (Med Associates, St. Albans, VT) were used for oxycodone self-administration. Each chamber was equipped with two response levers, a white cue light above each lever, a receptacle between the levers for delivery of food pellets (not used), and a house light for ambient illumination of the chamber. Each chamber was also equipped with a counterbalanced fluid swivel and tether, and an infusion pump (Model PHS-100, Med Associates, St. Albans, VT) that when activated delivered an infusion with a volume of 0.1 ml/kg in approximately 0.8 sec. Operation of apparatus and recording of data was controlled by MED-PC software and interfacing (Med-Associates, St. Albans, VT).

### 2.8 Oxycodone i.v. self-administration studies

A week after surgery, rats were trained to respond under a fixed-ratio (FR) 1 schedule in which each press of an active lever produced an infusion of 0.06 mg/kg i.v.. Sessions began with onset of the house light to signal availability of drug. Each infusion was signaled by offset of the houselight and onset of the cue light over the active lever during the infusion and a subsequent 6-sec timeout, during which responses were recorded but had no programmed consequence. Following the timeout, the cue light extinguished and the house light was illuminated to signal availability of the next unit dose. The training dose was chosen based on previous studies showing this dose produces reinforcing effects similar to heroin in rats [18]. Presses of the other (inactive) lever were recorded but had no programmed consequence. After ten sessions under the FR1 schedule, the FR value was increased to FR2 and FR3 for five sessions each. Rats were considered to have acquired self-administration if they earned at least 10 infusions per session and exhibited an active:inactive lever press ratio of at least 2∶1 during the last three sessions at FR3. Sessions were 120 minutes in duration and ran five days per week.

### 2.9 Brain collection

At 24 hours after the last oxycodone self-administration session, rats were rendered unconscious using CO_2_ chambers. Rats were decapitated using a guillotine and brains were removed, quickly dipped in isopentane kept in dry ice, wrapped in aluminum foil and frozen at −80°C prior to gene expression analysis.

### 2.10 Quantitative real-time reverse transcriptase polymerase chain reaction (qRT-PCR)

In this study, we analyzed mRNA expression of dynorphin, kappa opioid receptor (KOR), Gαi1, RGS9a, adenylate cyclase 5 (Adcy5), early growth response protein 2 (Egr2) and the early immediate gene c-Fos, which were found altered after chronic opioid administration in rats [27], [28].

Frozen brains (−80°C) were coronally sectioned on a cryostat (HM 505 E; Microm, Walldorf, Germany) until the rostral nucleus accumbens (NAc) and dorsal striatum (DStr) were exposed (Bregma 2.20 mm). Bilateral tissue punches 1 mm in length were taken of the NAc (diameter: 1 mm) and CPu (diameter: 2 mm) and placed in Eppendorf tubes kept on dry ice. RNA was extracted using PureLink RNA Mini Kit (Invitrogen) and quantified using a Nanodrop 2000 (Thermo Scientific). Total RNA (0.5 µg) was used to synthesize cDNA using iScript cDNA Synthesis Kit (BioRad) in a ThermoHybaid iCycler (Thermo Scientific). Primers specific for dynorphin (Dyn; Forward primer: CGCAAATACCCCAAGAGGAG, Reverse Primer: GCAGGAAGCCCCCATAGC; product size, 109 bp), kappa opioid receptor (KOR; Forward primer: CTCCCAGTGCTTGCCTACTC, Reverse Primer: AGATGTTGGTTGCGGTCTTC; product size, 240 bp), adenylate cyclase 5 (Adcy5; Forward primer: CCTCAACGACTCCACCTATG, Reverse primer: AATGACCCCAGCCACTACAG; product size, 166 bp), Gαi1 (Forward primer: TTTGGAGACGCTGCTCGTGCG, Reverse primer: GTACGCCGCCGAATCGTTCA; product size, 180 bp), Gαi2 (Forward primer: GATGATCGACAAGAACCTGCGGG, Reverse Primer: CAGGTTGCCCATGGCTTTGACG; product size, 223 bp), regulator of G-protein signaling 9 (Rgs9; Forward primer: AACAGCTGCCCAGGCTGAAACC, Reverse primer: TTGAGAGCAAGTGTGCCCCAGG; product size, 255 bp), Fos (Fos; Forward primer: CGGGAGTGGTGAAGACCATGTCAGG, Reverse primer: TCCGCTTGGAGCGTATCTGTCAGC; product size, 178 bp), early growth response 2 (Egr2; Forward primer: TCCCCAATGGTGAACTGGGAGG, Reverse primer: AGATGGGAGCGAAGCTACTCGG; product size, 132 bp), tubulin, alpha 1A (Tuba1a; Forward primer: AGAAGCAACACCTCCTCCTCGC; Reverse primer: AGGCTGGATGCCATGTTCCAGG; product size, 154 bp), integral membrane protein 2B (Itm2B; Forward primer: CATCCTGGGAGGAGCATACC; Reverse primer: TCCGCAAACTCTGGTACAGG; product size, 213 bp), and general transcription factor 2B (Gtf2B; Forward primer: TGCGATAGCTTCTGCTTGTC; Reverse primer: TCAGATCCACGCTCGTCTC; product size, 155 bp) genes were designed using NCBI Primer-BLAST (http://www.ncbi.nlm.nih.gov/tools/primer-blast/) and purchased from Integrated DNA Technologies (Coralville, Iowa). Melt curve analysis and polyacrylamide gel electrophoresis confirmed the specificity of the primers.

A Q-PCR kit (iQ SybrGreen Supermix, BioRad) was used. The qRT-PCR reaction was carried out using iQ SybrGreen Supermix (BioRad) on a MyiQ Single Color Real-Time PCR Detection System (BioRad) in a volume of 20 µl, with 2 µl of 3 mM forward and reverse primers and 4 µl cDNA sample diluted 1∶10. PCR cycling conditions were 95°C for 5 min; 40 cycles at 94°C for 15 s, 60°C for 15 s, 72°C for 15 s. Data were collected at read temperatures of 83–88°C for 15 s depending on amplicon melt temperatures. Standard dilution curves were generated for each primer set by serially diluting (1.00, 0.25, 0.0625, and 0.0156-fold) a master cDNA stock comprising an equal mix of cDNA from all treatment groups for a given brain region. The log_10_ of the dilution values was plotted against the threshold cycle values to generate standard curves. MyiQ Optical System Software (BioRad) was used to analyze the data. Samples containing no cDNA template and samples from cDNA synthesis reactions that contained no reverse transcriptase (no RT) were run as controls for contamination and amplification of genomic DNA, respectively. Reported values were normalized to the average values of the internal standards Tbp, Gtf2B, and Itm2B for each sample. Data are expressed as mean relative levels of the gene of interest/internal standards mRNA±SEM.

### 2.11 Statistical analysis

Mean daily infusions and responses on the active and inactive levers were analyzed via two-way ANOVA with treatment as a non-repeated factor and day as a repeated factor. Post-hoc comparisons between groups were done using unpaired t-tests and the False Discovery Rate (FDR), procedure with the FDR = 5% [29]. Mean infusions at FR3 (last three sessions), total infusions and total oxycodone intake were compared between vaccinated and control groups using unpaired t-tests, using Welch's correction where appropriate. The proportion of rats that met acquisition criteria was compared between groups using a Chi-square test. Quantitative RT-PCR data from each brain region were analyzed with two-way ANOVA, with treatment as a non-repeated factor and gene as a repeated factor. Significant effects between the OXY-KLH vaccine and the unconjugated KLH control groups were followed by Fisher's Least Significant Difference (LSD) post-hoc tests.

## Results

### 3.1 Vaccination with OXY-KLH attenuates acquisition of oxycodone i.v. self-administration

Vaccination with OXY-KLH resulted in oxycodone-specific serum IgG antibody concentrations of 450±65 µg/ml (mean±SEM), which are consistent with antibody concentrations previously observed in rats vaccinated with a heroin vaccine containing an identical linker and KLH as carrier [10], [25]. Compared to control rats immunized with the unconjugated KLH, vaccination with OXY-KLH reduced acquisition of oxycodone self-administration by lowering active lever pressing, number of infusions per session, overall oxycodone intake and also reduced the proportion of rats meeting acquisition criteria.

**Figure 1:**
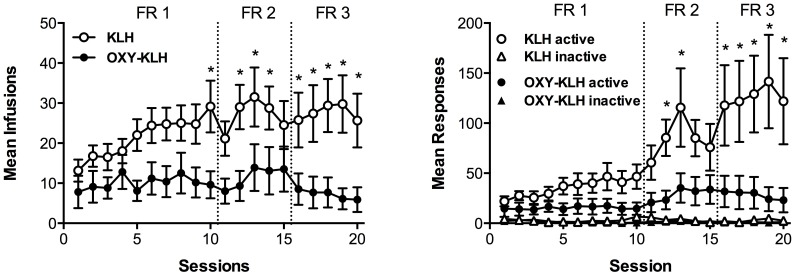
Vaccination attenuated acquisition of oxycodone self-administration in rats. Mean infusions of oxycodone (left panel) and mean responses on the active and inactive levers (right panel) in rats immunized with OXY-KLH (n = 10) or the unconjugated KLH carrier (n = 8). The fixed ratio (FR) is the number of active lever presses to deliver an intravenous infusion of 0.06 mg/kg oxycodone. All rats were tested in daily 120-minute sessions. Data are expressed as mean±SEM. Statistical symbols: *p<0.01 compared to the OXY-KLH group.

Figure 1 shows the mean number of infusions earned (left panel) and mean number of responses on the active and inactive levers (right panel) per session. Table 1 shows a summary of the findings for antibody titers and other measures of oxycodone self-administration. Analysis of response rate data showed significant main effects of treatment (F_(1,16)_ = 6.127, p<0.05) and day (F_(19, 304)_ = 8.125, p<0.0001), and a significant treatment x day interaction (F_(19, 304)_ = 4.409, p<0.0001). Compared to KLH controls, OXY-KLH vaccinated rats exhibited significantly lower rates of active lever pressing overall. Analysis of infusion data showed significant main effects of treatment (F_(1,16)_ = 6.616, p<0.05) and day (F_(19, 304)_ = 2.791, p<0.001), and a significant treatment x day interaction (F_(19, 304)_ = 2.34, p<0.01). Compared to controls, vaccinated rats exhibited significantly fewer mean infusions per session overall. By the end of the protocol (last three sessions at FR3), KLH control rats self-administered 28.25±6.689 infusions (mean±SEM), compared to only 6.567±3.092 infusions in vaccinated rats (t_(16)_ = 3.152, p<0.05), resulting in a significant difference in mean oxycodone intake (1.7±0.4 mg/kg vs 0.4±0.2 mg/kg, respectively, t_(10)_ = 2.94, p<0.05). During the entire study, control rats earned a mean total of 487.1±92.8 SEM infusions, while vaccinated rats earned a significantly lower mean total of 194.2±69.95 SEM infusions (t_(16)_ = 2.572, p<0.05), resulting in a significant decrease in cumulative oxycodone intake during the entire protocol (29.23±5.566 SEM mg/kg vs 11.65±4.197 SEM mg/kg, respectively, t_(16)_ = 2.572, p<0.05). Finally, the proportion of rats meeting acquisition criteria in the vaccine group was significantly lower compared to the control group, respectively 30 and 87.5% (*X* ^2^_(1)_ = 5.951, p<0.05).

**Table 1:** Oxycodone-specific serum IgG antibody titers are expressed as x 10^3^.

Treatment	Titers	Mean infusions (FR3)	Total infusions (all sessions)	Total drug intake (oxycodone, mg/kg)
KLH	n/a	28±7	487±93	30±6
OXY-KLH	327±64	7±3**	194±70*	12±4*

Data are expressed as mean± SEM.

*p<0.05,

**p<0.01 compared to KLH control.

### 3.2 Vaccination with OXY-KLH attenuates effects of oxycodone self-administration on adenylate cyclase 5, c-Fos, and early growth response 2 mRNA expression in rat striatum

**Figure 2:**
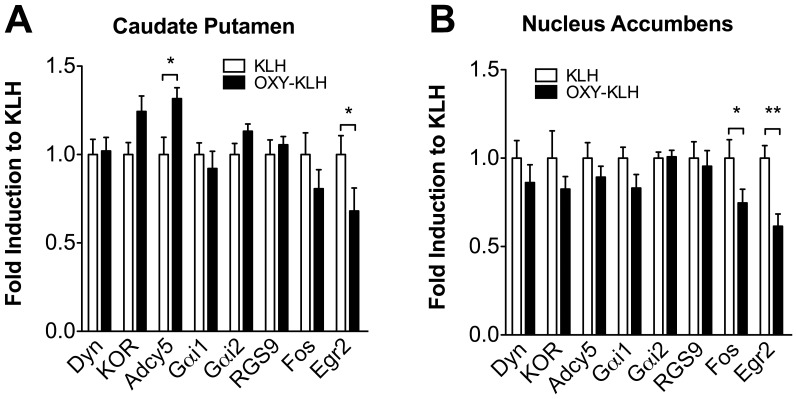
Vaccination effects on oxycodone-induced brain gene expression in rat striatum. Brain gene expression was analyzed by qRT-PCR at 24 hrs after the last oxycodone self-administration session. Panel A) caudate putamen, and panel B) nucleus accumbens. Data are expressed as mean±SEM. Statistical symbols: *p<0.05, **p<0.01 compared to rats immunized with unconjugated KLH control vaccination group.

At 24 hours after the last oxycodone self-administration session, we tested if vaccination affected the expression of several genes, previously shown to be regulated by chronic opiate treatment, including Adcy5, Egr2, c-Fos, Gαi1, and Rgs9. To this end, rats vaccinated with OXY-KLH were compared to KLH control rats using qRT-PCR on RNA extracted from the NAc and DStr. The effect of vaccination on gene expression in the NAc depended on treatment (F_(1,18)_ = 12.77, p<0.05) and in the DStr, it depended on an interaction between treatment and gene (F_(4,72)_ = 3.50, p<0.05). Post hoc tests revealed that Fos and Egr2 mRNA levels were significantly decreased (p<0.05) in the NAc of OXY-KLH rats compared to control rats immunized with unconjugated KLH. In the DStr, rats vaccinated with OXY-KLH showed significantly (p<0.05) more Adcy5 and less Fos compared to the KLH control group (Figure 2). These data suggest that oxycodone self-administration decreases Adcy5 expression and increases Fos and Egr2 expression, which is consistent with previous reports showing similar regulation of these genes in response to chronic morphine (Adcy5, Fos) [28] or heroin self-administration (Egr2, Fos) [27].

**Figure 3:**
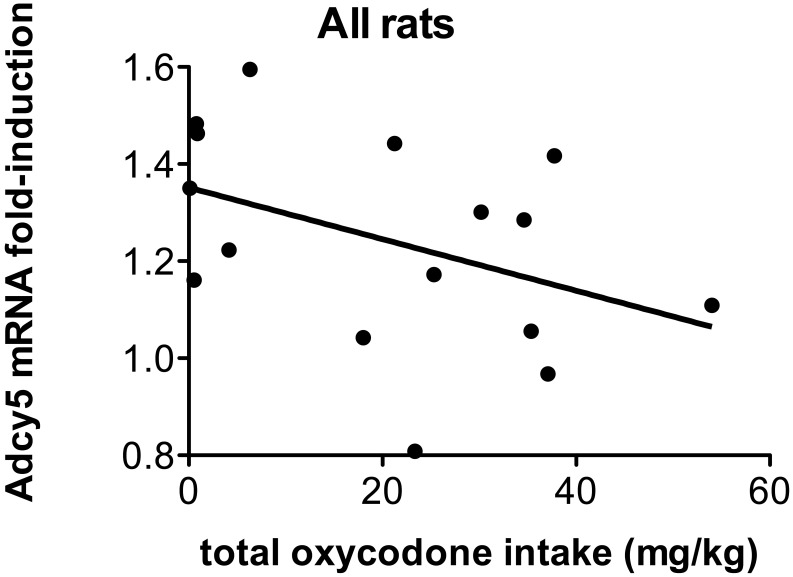
Relationship between Adcy5 mRNA expression and oxycodone overall dose intake. Linear regression of Adcy5 mRNA expression in the Caudate Putamen and oxycodone total dose intake (mg/kg) during the entire self-administration protocol. Total dose was calculated as the number of total number of infusions x infusion dose (0.06 mg/kg/inf). Data include all control (KLH) and vaccinated (OXY-KLH) rats.

To better understand the efficacy of vaccination in preventing oxycodone-induced changes in gene expression, we tested if there was any linear relationship between adenylate cyclase 5 mRNA expression in the CPu and oxycodone-specific antibody titers, mean infusions at FR3, total infusions and total oxycodone intake in rats vaccinated with OXY-KLH or in all rats. No relationship was found between Adcy5 mRNA expression and serum antibody titers, self-administration performance or oxycodone intake in vaccinated rats (not shown). However, among all rats, there was a trend showing that Adcy5 mRNA expression was reduced in rats with higher intake of oxycodone (Figure 3).

## Discussion

The main findings of this study were that compared to a control group immunized with the unconjugated carrier protein: 1) vaccination with an oxycodone conjugate vaccine reduced the proportion of rats that acquired oxycodone self-administration, 2) vaccinated rats showed decreased mean infusions and total oxycodone intake, and 3) 24 hours after 20 days of oxycodone self-administration, vaccinated rats showed altered mRNA expression in the brain reward pathway. The finding that vaccination blocked acquisition of oxycodone self-administration is consistent with previous reports showing that active or passive immunization can suppress nicotine, cocaine, heroin, or methamphetamine self-administration in rodents [7], [9], [11], [26], [30], [31], and extends this potential treatment approach to a class of abused drugs that are of great current concern.

Prior studies showed that vaccination with OXY-KLH reduces the early distribution of oxycodone and hydrocodone to the brain and attenuates their nociceptive effects in mice and rats [14], [15], [17], [25]. Additionally, OXY-KLH showed equivalent pre-clinical efficacy when administered by different routes or absorbed on different adjuvants, and vaccine efficacy was also preserved when the oxycodone-based 6OXY hapten was conjugated to the tetanus toxoid and GMP-grade KLH carriers suitable for human use [16]. The current study shows that OXY-KLH also markedly reduces oxycodone reinforcement in a key animal model of addiction. The total oxycodone dose self-administered per session in control rats was within the range of oxycodone doses that are commonly abused. The observed decrease in number of vaccinated rats acquiring oxycodone self-administration was similar to that of a functional 6-acetlymorphine conjugate vaccine for preventing acquisition of heroin self-administration [11], [12].

There was no relationship between the oxycodone-specific antibody titers and the observed reduction in mean infusions. This observation has been previously reported with nicotine vaccines, where no significant correlation was found between serum antibody titers and mean nicotine infusions [26]. This is somewhat surprising since it is known that immunization prevents drug distribution to the brain through various pharmacokinetic mechanisms. Possibly the sample size was too small to detect this relationship or other sources of variability obscured it.

In the current study, during 120-minute intravenous self-administration sessions at a FR3 schedule of reinforcement, the total mean oxycodone intake in KLH control rats was 1.7±0.4 mg/kg (mean±SEM). In previous studies, vaccination with OXY-KLH decreased the distribution of oxycodone to the brain by 51% at 5 minutes after i.v. administration of 0.5 mg/kg and by 74% at 30 minutes after s.c. administration of 2.25 mg/kg in rats [14], [15]. These doses, 0.5 mg/kg i.v. and 2.25 mg/kg s.c., produced respectively serum oxycodone concentrations between 82±22 and 428±179 ng/ml (mean±SD) [14], [15]. In healthy subjects, oral doses of 10–15 mg of instant-release oxycodone resulted in peak plasma concentrations of 15–20 ng/ml at 60–90 minutes after administration [32], [33]. These data suggest that OXY-KLH may be able to prevent distribution of clinically-relevant doses of oxycodone.

In clinical studies of a nicotine vaccine, it has been shown that vaccination reduced occupancy of β_2_-containing acetylcholine nicotinic receptors in cortical structures of smokers receiving i.v. bolus nicotine [34]. The present study shows that vaccination is sufficient to prevent or alter drug-induced changes in mRNA expression in subjects chronically exposed to oxycodone. Vaccination prevented oxycodone-induced down-regulation of Adcy5 mRNA in the dorsal striatum, which occurs after chronic opioid exposure [28]. In control rats immunized with the KLH carrier alone, Adcy5 expression was reduced compared to rats vaccinated with OXY-KLH suggesting that oxycodone self-administration, or chronic exposure to oxycodone, down-regulates the expression of Adcy5 in the dorsal striatum and that vaccination may prevent it. In vaccinated rats, Egr2 and the immediate early gene c-Fos were down-regulated compared to the KLH control group, suggesting that vaccination may prevent increases of striatal Egr2 and Fos expression in rats chronically exposed to opioids. These findings indicate that vaccination prevented several drug-induced gene expression changes commonly associated with opioid dependence or chronic opioid exposure.

It is likely that the effects of OXY-KLH on mRNA expression were the consequence of vaccination reducing the amount of oxycodone that reached the brain during each self-administration session. In support of this, there was a trend toward a relationship between the amount of oxycodone self-administered and Adcy5 mRNA expression. This hypothesis is further supported by a correlation found between the overall oxycodone intake during self-administration and the mRNA expression of the neurotransmitter Monoamine Oxidase A in mice [21]. The relationship between oxycodone exposure during self-administration studies and changes in brain gene expression in the striatum may have also been mediated by oxycodone-induced neurochemical changes. Since it has been shown that oxycodone self-administration increases striatal dopamine release [22], it would be important to test if vaccination against oxycodone has the ability of preventing oxycodone-induced dopamine modulation within the mesolimbic system.

In this study, no effect of vaccination was detected in the dynorphin, KOR, Gαi1, Gαi2 and RGS9 mRNA expression in either NAc or CPu. During exposure to opioids, Adcy5 activity in the striatum is tightly regulated by RGS9 modifying the kinetics of the Gαi1 subunit and Gβγ complex in opioid G-protein coupled receptors [35]. It was therefore surprising to find no differences in expression of Gαi1, Gαi2 and RGS9. The lack of changes in mRNA expression may be due to the effect of chronic exposure to oxycodone, which may mask effects otherwise detected after acute opioid administration. Additionally, drug exposure may result in alteration of enzymes, protein or activity rather than changes in gene expression, which may explain why no oxycodone-induced changes in Gαi1, Gαi2 and RGS9 expression were detected in this study.

Overall, these data add to the evidence supporting the preclinical efficacy of vaccination with OXY-KLH in blocking oxycodone-induced addiction relevant behaviors, and support its potential role in treating oxycodone abuse. Hydrocodone self-administration was not studied, but OXY-KLH is effective in reducing hydrocodone distribution to brain and antinociception in rats, to the same degree as it blocks oxycodone effects. This study also provides preliminary evidence of the potential ability of vaccination to alter the effects of oxycodone on several key neural mediators of opioid behavioral effects.
